# Identification of Clinical Phenotypes Using Cluster Analyses in COPD Patients with Multiple Comorbidities

**DOI:** 10.1155/2014/420134

**Published:** 2014-02-10

**Authors:** Pierre-Régis Burgel, Jean-Louis Paillasseur, Nicolas Roche

**Affiliations:** ^1^Service de Pneumologie, Hôpital Cochin, Assistance Publique Hôpitaux de Paris, 27 rue du Faubourg St. Jacques, 75014 Paris, France; ^2^Université Paris Descartes, Sorbonne Paris Cité, 75014 Paris, France; ^3^Initiatives BPCO Study Group, France; ^4^EFFI-STAT, 75004 Paris, France

## Abstract

Chronic obstructive pulmonary disease (COPD) is characterized by persistent airflow limitation, the severity of which is assessed using forced expiratory volume in 1 sec (FEV_1_, % predicted). Cohort studies have confirmed that COPD patients with similar levels of airflow limitation showed marked heterogeneity in clinical manifestations and outcomes. Chronic coexisting diseases, also called comorbidities, are highly prevalent in COPD patients and likely contribute to this heterogeneity. In recent years, investigators have used innovative statistical methods (e.g., cluster analyses) to examine the hypothesis that subgroups of COPD patients sharing clinically relevant characteristics (phenotypes) can be identified. The objectives of the present paper are to review recent studies that have used cluster analyses for defining phenotypes in observational cohorts of COPD patients. Strengths and weaknesses of these statistical approaches are briefly described. Description of the phenotypes that were reasonably reproducible across studies and received prospective validation in at least one study is provided, with a special focus on differences in age and comorbidities (including cardiovascular diseases). Finally, gaps in current knowledge are described, leading to proposals for future studies.

## 1. Introduction

Chronic obstructive pulmonary disease (COPD) is characterized by incompletely reversible airflow limitation and its prevalence is strongly associated with ageing and smoking [1]. It has long been noticed that COPD patients constitute a heterogeneous group of patients and a large observational study recently confirmed that subjects with similar range of airflow limitation (as defined by FEV_1_% predicted) had marked differences in symptoms (dyspnea, cough, and sputum production), rates of exacerbations, exercise capacity, and health status [2]. Based on these findings, interest has grown on the identification of subgroups of COPD patients sharing clinically meaningful characteristics, also called phenotypes [3].

In the past decade, it has become clear that chronic diseases often coexist [4]. Accordingly, COPD patients often suffer from other chronic diseases (called comorbidities). Cardiovascular diseases, psychological disorders (e.g., anxiety and/or depression), metabolic disorders (e.g., diabetes, dyslipidemia, and metabolic syndrome), and cancer have been found highly prevalent among COPD patients [5, 6]. The association of comorbidities and COPD may merely reflect common risk factors (e.g., age or cigarette smoking). However, chronic low-grade systemic inflammation, which is observed in some COPD patients, could also be involved [7], as well as biological mechanisms related to decreased physical activity, which is often observed with ageing [8]. The importance of coexisting diseases has been underscored by several studies showing that COPD patients with multiple comorbidities have worse prognosis [4, 9–11]. However, it has not been established whether the presence of one or several of these comorbidities represent or contribute to a coherent phenotype per se.

Recently, several groups have reported studies aimed at finding COPD phenotypes using multivariable exploratory analyses (e.g., cluster analysis). The purpose of the present paper is to review recent evidence obtained from studies that have searched for COPD phenotypes using cluster analyses of observational data obtained in COPD cohorts, with a special interest in the contribution of comorbidities to phenotypes.

## 2. COPD Phenotypes: A Historical Perspective

Historically, the concept of COPD phenotypes probably goes back to the recognition of two major COPD components, that is, emphysema and chronic bronchitis [12]. These components had been described long before the first appearance of the term COPD in 1960 [13], but they had been considered as separate diseases before being unified under the term COPD. Interestingly, as soon as the use of the term COPD began being generalized, the disease was recognized as being markedly heterogeneous [14]. At the 9th Aspen Emphysema Conference, Burrows and Fletcher presented their findings on “the emphysematous and bronchial types of chronic airway obstruction,” which were subsequently published in 1968 [15]. The authors defined two distinct aspects (emphysema, type A or “pink puffer” and airways disease, type B or “blue bloater”) of a single condition (chronic airflow obstruction), being a precursor of the phenotypes concept in the COPD area. Almost half a century later, the 48th Conference (renamed “Thomas L. Petty Aspen Lung Conference”) began with a clinical theme emphasizing clinical COPD phenotypes [16]. In the meantime, reference to A and B types of chronic airway obstruction had disappeared, due to the lack of relation between clinically relevant outcomes and pathologic findings, especially regarding the centri- or panlobular nature of emphysema: the traditional definition of phenotypes (referring to “the observable structural and functional characteristics of an organism determined by its genotype and modulated by its environment” [17]) was already considered insufficient to establish clinically relevant COPD phenotypes. Therefore, Han et al. recently proposed a novel definition of COPD phenotypes, that is, ‘‘a single or combination of disease attributes that describe differences between individuals with COPD as they relate to clinically meaningful outcomes (symptoms, exacerbations, response to therapy, rate of disease progression, or death)” [3].

## 3. Relation of Phenotypes to Prognostic Indices and COPD Classifications

As suggested in a recent editorial [18], it seems important to avoid confusion between phenotypes and markers of disease severity (e.g., prognostic indices) or disease activity. Additionally, the recent update of the Global Initiative for Obstructive Lung Disease (GOLD) has introduced a new multidimensional classification of COPD, with four categories that could correspond to phenotypes.

### 3.1. Prognostic Indices Do Not Identify Phenotypes

Several multidimensional prognostic indices have been developed in COPD [19], the BODE index being one of the most widely quoted [20]. By definition, these tools were shown to reliably predict the risk of death and/or other outcomes such as the risk of exacerbation or hospitalization, at least in the populations in which they were developed. For many of them, external validity was also demonstrated, even if some adjustments “recalibration” were sometimes advocated. Importantly, patients who share similar prognosis (at least from a statistical, population perspective) based on a similar prognostic score might not necessarily be considered as belonging to a specific phenotype. For example, an 80-year-old patient with a body mass index (BMI) at 20 kg/m^2^, medical research council (MRC) dyspnea grade 3, FEV_1_ 45% predicted, and 6-min walking distance (6MWD) = 340 m will have the same BODE score (i.e., 6) as a 55-year-old patient with FEV_1_ 28% predicted, BMI 28 kg/m^2^, MRC grade 3, and 6MWD = 255 m. Although sharing the same predicted survival, these two patients look very different and will certainly not die at the same age. Thus, it seems difficult to state that they belong to the same phenotype, and prognostic indices are not substitutes for phenotypes.

### 3.2. COPD Classifications: From Lung Function to Multidimensional Assessment

Until recently, COPD classification was largely based on FEV_1_ [21], which was poorly correlated with symptoms [2]. Even if guidelines advocated the need for thorough clinical assessment of symptoms and exacerbations, they did not formalize the way these items had to be accounted for. In 2011, the GOLD classification was profoundly modified. It now comprises four quadrants (A-B-C-D) based on (i) symptoms (dyspnea and health status/global impact) and (ii) risk of exacerbations estimated through the severity of airflow obstruction (with the same grades 1-2-3-4 as previously stated) and previous history of exacerbations/hospitalizations [22]. Descriptions of the four groups look like phenotypes: low symptoms/low risk, high symptoms/low risk, high risk/low symptoms, and high risk/high symptoms. However, it must be outlined that, although associated with several differences in clinical characteristics and prognosis [23], these four categories are the result of expert opinions rather than formal statistical approaches. In addition, how they predict mortality is debated (with some discrepancies between studies, and a discriminant capacity that does not exceed that of FEV_1_-based classification), and their relations with response to treatments are unknown. Finally, age and comorbidities were not included in this novel classification, suggesting that they account only partially for the heterogeneity of COPD patients.

## 4. Statistical Strategies for Identifying COPD Phenotypes

Disease characteristics that could be used to define phenotypes of patients with chronic airway diseases include clinical features (e.g., risk factors, clinical manifestations, and comorbidities), imaging (e.g., emphysema, airway thickening, and bronchiectasis), pulmonary function and exercise tests, and biomarkers. Integrating these various characteristics with the aim of defining phenotypes is challenging. Historically, investigators have used classical multivariable analyses, but recent studies have highlighted the potential of using cluster analyses for this purpose.

### 4.1. Classical Multivariable Analysis

The classical approach to the identification of COPD phenotypes seeks associations between phenotypic characteristics (also called phenotypic traits) and outcomes using multivariable analyses (i.e., logistic or multilinear regression analyses). For example, sputum or bronchoalveolar lavage eosinophilic inflammation has been associated with response to systemic corticosteroids in COPD patients [24] and the presence of chronic cough and sputum production has been associated with poorer long-term outcomes in terms of lung function decline [25] and risk of exacerbations and hospitalizations [26]. Further, repeated hospitalizations were independently associated with mortality in COPD patients [27]. All these studies related a single disease attribute, usually identified by physicians based on observation, to a specific outcome. However, classical statistical analyses can also be used for phenotypic characterization using combinations of disease attributes: for example, investigators of the National Emphysema Treatment Trial found that COPD patients with emphysema who have a low FEV_1_ and either homogeneous emphysema or a very low carbon monoxide diffusing capacity were at high risk for death after lung volume reduction surgery and also were unlikely to benefit from the surgery [28].

Although this classical statistical approach has produced interesting results, leading to the identification of potential COPD phenotypes including those described above and others (e.g., frequent exacerbators [29]), it is usually based on clinical observation of a limited number of variables and may have missed more complex phenotypes. Because integrating the growing number of information available in clinical medicine only using clinical judgment may be difficult, it was suggested that mathematical models may help in unraveling the complexity of COPD [30].

### 4.2. Cluster Analyses and Related Exploratory Analyses

The term “cluster analysis” refers to a group of statistical methods that seek to organize information so that data from heterogeneous variables can be classified into relatively homogeneous groups [30]. In recent years, cluster analysis has been used to examine heterogeneity of patients with chronic airway diseases including asthma [31, 32] and COPD [33–40]. Cluster analysis is often presented as an unsupervised and unbiased method. However, important aspects related to the different methods of cluster analysis and to the choice of variables included in the analysis may affect the results.

The two main different cluster analyses methods, which have been used in studies of airway diseases, are hierarchical and nonhierarchical (e.g., K-means) cluster analyses. Hierarchical cluster analysis is based on the idea that patients who share similarities on a set of data can be grouped together. In the agglomerative techniques (the most widely used), the results are shown as a dendrogram in which each horizontal line represents an individual subject and the length of horizontal lines represents the degree of similarity between subjects [30, 41]. The number of clusters is determined according to the results of the analysis (see below). In K-means cluster analysis, the number of clusters (*k*) is determined before the analysis and the algorithms find the cluster center and assign the objects to the nearest cluster center [30, 41]. Drawbacks of the K-means analysis are the necessity of choosing the *k* number of clusters and the fact that the algorithms usually prefer clusters of approximately similar size, which may lead to ignoring a smaller, yet important, group of subjects [30, 41]. Self-organizing maps (SOM) are an alternative neural network-based nonhierarchical clustering approach, which has been used for analysis of gene arrays [42] and has also been used recently to examine comorbidities in COPD patients [40].

The choice of variables included in a cluster analysis is a very important aspect of the analysis; cluster analysis detects structures within selected variables, but cannot determine whether some of the selected variables are irrelevant for phenotyping. The choice of variable is dictated by practical considerations (e.g., the type of data available in the cohort), underscoring the need for well-characterized cohorts. Although some investigators performed cluster analysis with as many variables as available in their database [37], we believe that selection of a limited number of variables considered important in defining the disease process may be preferable. For example, when looking for phenotypes associated with different risk of mortality, our cluster analyses were based on data previously associated with death in COPD patients [34, 43]. This strategy is very similar to what has been performed for analysis of multiple genes using gene arrays: although it is possible to set up arrays using very large numbers of gene, it is also possible to use gene sets containing smaller numbers of genes that are defined based on prior biological knowledge, for example, published information about biochemical pathways [44].

The encoding of variables (e.g., using raw data, categorized data, or *Z*-scores [37]) may also affect the results of a cluster analysis [45], but this aspect has not been formally explored in the COPD literature. Further, the ways to handle missing data, which are present in any large dataset of observational data, have been different among studies; it has resulted in exclusion of patients from the analysis in some studies [34, 35], whereas other investigators suggested the usefulness of multiple imputation for missing data, to avoid excluding patients from the analyses [46]. The impact of patient exclusion versus imputation remains to be established.

Another important aspect relates to the fact that correlations between initially selected variables may add statistical noise and corrupt the cluster structure. To limit this problem, strategies of data transformation using principal component analysis (for numerical variables) and/or multiple correspondence analyses (for categorical variables) have been proposed [26, 43]. An advantage of using these techniques is the ability to combine mathematical axes obtained in these analyses in a single cluster analysis, allowing analyzing of numerical and categorical variables simultaneously [43]. However, when studying a limited number of continuous or categorical variables that are not closely correlated with each other, these steps of data transformation may not be necessary.

Choosing the appropriate number of clusters among the multiple possibilities generated by the analyses may also be challenging. In hierarchical cluster analysis, the number of clusters can be deducted from visual inspection of the dendrogram, or from statistical measurement of large jumps in the similarity measure at each stage (e.g., pseudo-F and/or pseudo-T2 statistics) [34]. However, the number of clusters can also be deducted from the clinical outcome used for validation of the analysis. For example, in an analysis of the Leuven COPD cohorts, statistical methods based on the dendrogram obtained by hierarchical cluster analysis suggested that data could be optimally grouped in 3 or 5 clusters [43]. When grouping the data into 3 clusters, there was a clear difference in mortality rates among clusters, whereas grouping data into 5 clusters did not improve the ability to predict mortality, leading to the final choice of 3 clusters [43]. When using K-means clusters (or equivalent nonhierarchical cluster analyses), the number of clusters needs to be prespecified [37]. Although there are statistical methods to determine the optimal number of clusters (e.g., performing a hierarchical cluster analysis before the K-means analysis [31]), investigators have also used clinical judgment to determine the optimal numbers of prespecified clusters [37].

In summary, various strategies of data selection, data transformation, and use of various algorithms clearly underscoring that exploratory cluster analyses in airway have been used, diseases cannot be considered as really “unsupervised and unbiased.” Thus, cluster analysis should be better viewed as a supervised multivariable exploratory analysis, and its results need to be validated using clinically relevant endpoints in multiple cohorts of patients.

### 4.3. Limitations of Current Studies Aimed at Finding COPD Phenotypes Using Cluster Analyses

In recent years, several studies have used cluster analyses to examine cohorts of COPD patients aiming at the identification of clinical phenotypes in stable patients [33–40, 43]. In the present paper, we will not examine studies performed in mixed populations of patients with various chronic airway diseases [30, 47], in COPD patients recruited in clinical trials [48] (i.e., who may not be representative of the real-world COPD population), nor studies that aimed at the identification of phenotypes of COPD exacerbations [49].

A summary of studies that have used cluster analyses for identification of COPD phenotypes is presented in Table 1. Several limitations of these approaches should be acknowledged. First, all studies were performed in relatively small numbers of patients recruited either in a single center or in multiple centers in a single country. Their designs have likely resulted in the selection of patients that cannot be considered representative of the COPD population at large and thus may have missed important phenotypes; for example, Altenburg et al. [33] and Vanfleteren et al. [40] recruited patients participating in rehabilitation programs; Garcia-Aymerich et al. recruited patients at the time of their first hospitalization in Spain, and these patients were almost exclusively (93%) men [37]. Burgel et al. recruited patients who were all followed in tertiary care [34] or combined a cohort of patients followed in tertiary care with a cohort of milder COPD patients identified in a lung cancer screening study [35]. Fens et al. also studied patients with mild airflow limitation diagnosed during a lung cancer screening study [36].

Second, there was marked heterogeneity in the data selected for cluster analyses. Some studies selected only clinical data and pulmonary function tests, whereas others also included imaging and/or biological biomarkers; these choices, often based on the availability of data, could have affected the results. Regarding comorbidities, several studies did not report assessment of comorbidities in their patients [33, 38, 39]. Others examined self-reported [36, 37] or physician-diagnosed [34, 35] comorbidities, both of which may have resulted in underestimations due to the high level of undiagnosed comorbidities in COPD patients [50]. Only one study has performed systematic assessment of several comorbidities [40]. Of note, cluster analysis reported in this study was performed using data on the presence of comorbidities (categorical) and the degree of their presence (linear), but not using data characterizing COPD (e.g., pulmonary function tests) [40].

Finally, validation of possible phenotypes using longitudinal outcomes was performed only in a limited number of studies: Burgel et al. performed two studies in two different cohorts of COPD outpatients and validated their findings using all-cause mortality [34, 35, 43]. Garcia-Aymerich et al. studied patients recruited at the time of a first hospitalization for COPD exacerbation and used all-cause mortality and hospitalizations related to COPD and to cardiovascular diseases to validate their findings [37]. Altenburg et al. found two phenotypes in which patients responded differently to pulmonary rehabilitation [33]. Other studies did not report prospective validation of their findings. At the end, although these limitations should be taken into consideration, cluster analyses have resulted in interesting preliminary results that are summarized in the next section.

### 4.4. Main COPD Phenotypes Identified by Cluster Analyses

Several possible phenotypes were identified in the various studies that have used cluster analyses in observational cohorts of COPD patients (see Table 1). Here we limit the description to the phenotypes (i) that were reasonably reproducible across various studies performed in various countries, using various initial data sets and various types of cluster analyses and (ii) that received prospective validation in at least one study.

Several studies have identified groups of COPD subjects with metabolic and cardiovascular comorbidities. Garcia-Aymerich et al. identified a cluster of COPD patients with “systemic COPD” [37]. These subjects were characterized by a high body mass index and very high rates of diabetes, congestive heart failure and ischemic heart disease; interestingly, they had higher levels of dyspnea and poorer health status than subjects with comparable airflow limitation, but less cardiovascular and metabolic comorbidities [37]. Importantly, these patients were at high risk of hospitalization for cardiovascular events and also had substantial risk of hospitalization for COPD (despite having moderate airflow limitation) and all-cause mortality [37]. These findings were consistent with those of Burgel et al. who reported marked differences between two clusters of subjects with comparable moderate to severe airflow limitation [34]. Subjects with high rates of obesity, diabetes, and cardiovascular comorbidities had more symptoms and higher rates of exacerbations. Interestingly, these subjects were markedly older, a finding consistent with the increasing prevalence of cardiovascular diseases and obesity with age [51]. Vanfleteren et al. also found a group of subjects with cardiovascular comorbidities but mostly normal BMI and suggested that this group differed from another one called “metabolic” in whom subjects showed high rates of obesity, dyslipidemia atherosclerosis, and myocardial infarction [40]. Although it remains unclear whether COPD patients with cardiovascular versus metabolic comorbidities truly represent two different groups of patients, it is concluded that most studies identified these comorbidities in subsets of patients with worse prognosis compared to other COPD subjects.

Burgel et al. have identified subjects with severe airflow limitation occurring at an early age in two different cohorts of COPD patients [34, 43]. These subjects were characterized by nutritional depletion [34, 43], high rates of emphysema and COPD exacerbations [43], muscle weakness, and high rates of osteoporosis [43], but very low rates of cardiovascular comorbidities [34, 43]. In both studies, these subjects were at very high risk of mortality at a relatively young age [34, 43], suggesting that specific therapeutic intervention should be targeted to this group of very severe patients. Interestingly, Vanfleteren et al. also found a cluster of cachectic subjects who were very similar to these latter subjects [40]. These authors suggested that common pathophysiologic pathways may be responsible for the cooccurrence of emphysema, muscle wasting, and osteoporosis. Of note, women appeared most prevalent in the cachectic phenotype in all 3 studies [34, 40, 43], a finding that is consistent with data obtained in the Boston Early-Onset COPD study [52].

Finally, Vanfleteren et al. identified a cluster of subjects with less comorbidity [40]. Interestingly, COPD patients without significant rates of major comorbidities were also found in other studies [34, 35]. Although these data suggest that COPD may occur in the absence of other comorbidities, this absence may be interpreted differently in younger subjects (in whom comorbidities may occur later with ageing, if they survive long enough) and in older subjects (in whom these comorbidities are presumably less likely to occur as they did not in previous years). Nevertheless, at a similar level of FEV_1_, patients with less comorbidities were suggested to have less COPD exacerbations [34].

## 5. Future Studies and Implications

### 5.1. Future Studies

The studies described in this review paper have produced interesting results by showing the feasibility of using cluster analyses and associated statistical methods for unraveling the heterogeneity of COPD patients. As already explained, all the previously published studies had limitations, largely related to the settings of patient recruitment in these cohorts (see above). Large cohorts, containing detailed information on patients recruited in multiple settings, are costly to establish. One option could be to merge multiple cohorts obtained in different settings, to ensure representation of different subgroups of patients. In this regard, future analyses should consider grouping cohorts that recruited inpatients in tertiary care (which may contain the most severe patients, including those awaiting for lung transplantation) with cohorts of in/outpatients recruited in secondary care and cohorts of patients recruited in primary care. Additionally, inclusion of preclinical COPD patients (e.g., recruited through systematic screening in the community) will ensure that all groups of age, disease severity, gender, and other patients characteristics (e.g., comorbidities, risk factors, social background,…) will be represented. Further, obtaining data from various areas of the world will provide better representation of patients with various genetic backgrounds and environmental exposures and will account for differences in healthcare systems.

Although there is currently no consensus on which data are required for optimally phenotyping COPD patients, it appears clear that characterization of patients should not be limited to the respiratory system but should include comorbidities. Undiagnosed comorbidities are highly prevalent and may have an important impact on COPD patients, suggesting that systematic assessment of comorbidities may be preferable, although it may be difficult to achieve in large cohorts of patients.

Prospective validation of phenotypes using clinically meaningful endpoints appears mandatory [3]. Longitudinal followup of phenotypes will also be interesting for examining their stability, as all current studies were performed using transversal rather than longitudinal data. Although phenotype stability is a major problem in asthmatic patients [53], a disease characterized by marked variability, it is probably less problematic in COPD, especially in older patients in whom airflow limitation and comorbidities are unlikely to show marked changes with time. However, followup of younger COPD patients with less comorbidity will be interesting to examine whether or not ageing will result in incident comorbidities and in the progression of airflow limitation and COPD-related outcomes. Furthermore, researchers should concentrate on establishing physician-friendly rules for assigning patients to appropriate phenotypes in daily practice. Tree-diagram analysis has proven useful to assign asthmatic patients to cluster-defined phenotypes using easily available clinical data [32] and may provide interesting insight in COPD subjects.

### 5.2. Future Implications

Identification of clinical COPD phenotypes using cluster analyses may ultimately result in important changes in our conception of COPD. Validation of phenotypes across multiple cohorts of patients in various settings (see above) may result in the development of novel classifications of COPD patients, better reflecting their heterogeneity. Each phenotype may have different pathophysiology and identification of biological mechanisms specific of some phenotypes (endotypes) may lead to the development of biomarkers aimed at early diagnosis of phenotypes and identification of candidates to specific, more targeted treatments. From a methodological perspective, there are two ways to identify endotypes associated with phenotypes: the first is to identify clinical phenotypes first, then determine which biological mechanisms are associated with them; the second is to mix clinical and biological variables in cluster analyzes. Available data do not allow determining which approach is the most relevant, and they might be complementary.

Finally, we propose that classifying patients based on some of the phenotypes consistently identified in various cluster analyses may provide an interesting alternative to currently used criteria based mostly on FEV_1_, symptoms and, exacerbation history for recruiting patients in clinical trials. For example, selecting patients who also share other similar characteristics (e.g., age, presence or absence of comorbidities) and future risks may provide a form of enrichment strategy, allowing for smaller sample size and shorter duration of followup [54].

## Figures and Tables

**Table 1:** Summary of studies exploring possible phenotypes using cluster analyses in stable COPD patients.

Reference	*n*	Setting	Population characteristics	Data used to build clusters	Multiple comorbidities	Types of analyses	Main results	Outcome for validation
Altenburg et al. [33]	65	Single center, tertiary care, and pulmonary rehabilitation (Groningen, The Netherlands)	Moderate to very severe airflow limitation Referred for rehabilitation	Age, BMI, quadriceps force, body plethysmography, and exercise testing	Not assessed	K-means	2 phenotypes: (i) worse lung function and exercise capacity, worse quadriceps force, and better response to exercise training (ii) better lung function and exercise capacity and less response to exercise training	High or low improvement in endurance exercise capacity rehabilitation

Burgel et al. [34, 35]	322	Multicenter cohort (Initiatives BPCO), and tertiary care (France)	Mild to very severe airflow limitation Outpatients	Age, history, and symptoms, spirometry, BMI, exacerbations, health status, psychological status	Physician-diagnosed Not included in the cluster analysis	PCA, HCA (Ward's)	4 phenotypes: (i) young subjects with severe respiratory disease, cachexia (ii) older subjects with mild airflow limitation and mild comorbidities (iii) young subjects with moderate to severe airflow limitation, but few comorbidities (iv) older subjects with moderate to severe airflow limitation and high rates of cardiovascular comorbidities	All-cause mortality

Burgel et al. [43]	527	Single center, tertiary care (Leuven, Belgium)	Mild to very severe airflow limitation Outpatients and COPD patients identified as part of a lung cancer screening study	Age, history and symptoms, health status, body plethysmography, DL_CO_, CT-scan, and physician-diagnosed comorbidities	Physician-diagnosed Included in the cluster analysis	PCA, MCA, HCA (Ward's)	3 phenotypes: (i) younger patients with severe respiratory disease, cachexia, and low rates of cardiovascular comorbidities. (ii) older patients with less severe airflow limitation, but often obese and with high rates of cardiovascular comorbidities and diabetes. (iii) mild to moderate airflow limitation, absent or mild emphysema, absent or mild dyspnoea, normal nutritional status, and limited comorbidities	All-cause mortality

Fens et al. [36]	157	Population-based survey (Utrecht, The Netherlands)	Mild to moderate airflow limitation COPD patients identified as part of a lung cancer screening study	History and symptoms, health status, comorbidities, spirometry, DL_CO_, CT-scan, and breathomics (electronic nose)	Self-reported Included in the cluster analysis	PCA, HCA (Ward's), K-means	4 possible phenotypes: (i) mild COPD (ii) moderate airflow obstruction with chronic bronchitis and emphysema (iii) asymptomatic emphysema with preserved lung function (iv) high symptoms, preserved lung function	None

Garcia-Aymerich et al. [37]	342	Multicenter study, tertiary care (Spain)	Mild to very severe airflow limitation COPD patients recruited after a 1st hospitalization	History and symptoms, health status, body composition, body plethysmography, CT-scan, biology (sputum and serum), and exercise testing	Self-reported Included in the cluster analysis	K-means	3 phenotypes: (i) severe respiratory COPD (ii) moderate respiratory COPD (iii) systemic COPD (high rates of cardiovascular comorbidities)	(i) Hospitalizations (COPD or cardiovascular) (ii) all-cause mortality

Paoletti et al. [38]	415	Single center, tertiary care (Florence, Italy)	Mild to very severe airflow limitation Outpatients	History and symptoms, body plethysmography, DL_CO_, and chest X-ray	Not assessed	MDS, PCA, MCA, K-means	2 phenotypes: (i) predominant airflow obstruction (ii) predominant parenchymal destruction	None

Pistolesi et al. [39]	322	Single center, tertiary care (Florence, Italy)	Mild to very severe airflow limitation Outpatients	History and symptoms, body plethysmography, DL_CO_, and chest X-ray	Not assessed	MDS, PCA, cluster analysis*	2 phenotypes: (i) predominant airflow obstruction (ii) predominant parenchymal destruction	None

Vanfleteren et al. [40]	213	Single center, tertiary care, pulmonary rehabilitation (Horn, The Netherlands)	Moderate to very severe airflow limitation Referred for rehabilitation	13 comorbidities	Systematically assessed Cluster analysis performed exclusively on comorbidities	SOM, HCA (Ward's)	5 possible comorbid phenotypes: (i) less comorbidity (ii) cardiovascular (iii) cachectic (iv) metabolic (v) psychological with no difference in systemic inflammation	None

*Type of cluster analysis not described; HCA: hierarchical cluster analysis; PCA: principal component analysis; MCA: multiple correspondence analysis; MDS: multidimensional scaling; SOM: self-organizing maps.
